# Artificial Sweetener Use in Hungary: A Cross-Sectional Study on Socioeconomic and Health Disparities from a Public Health Perspective

**DOI:** 10.3390/nu17142352

**Published:** 2025-07-17

**Authors:** Battamir Ulambayar, Marianna Móré, Attila Csaba Nagy

**Affiliations:** 1 Department of Health Informatics, Faculty of Health Sciences, University of Debrecen, 4032 Debrecen, Hungary; ulambayar.battamir@etk.unideb.hu; 2 Institute of Social and Sociological Sciences, Faculty of Health Sciences, University of Debrecen, 4400 Nyiregyhaza, Hungary; more.mariann@etk.unideb.hu

**Keywords:** artificial sweetener use, public health, Hungary, European health interview survey

## Abstract

**Background/Objectives**: The use of artificial sweeteners (AS) is increasing globally despite growing evidence suggesting potential health risks. This study investigates the sociodemographic and health-related factors associated with AS use in the Hungarian population. **Methods**: We conducted a cross-sectional analysis using data from the 2019 European Health Interview Survey (EHIS), comprising 5603 participants. AS users were identified based on self-reported use of AS. Logistic regression models were used to examine associations between regular AS use and demographic, socioeconomic, and health variables. Interaction terms were included to explore potential effect modification. **Results**: AS use was reported by 20.1% of participants. Older adults, individuals with overweight or obesity, and those reporting poorer self-perceived health were more likely to use AS. AS use was also higher among individuals in lower and middle-income quintiles. Interaction analyses revealed that overweight and obese individuals with the lowest income, as well as older adults in poor health, were particularly likely to use AS. **Conclusions**: The findings highlight disparities in AS use across age, income, BMI, and health status, raising concerns about the public’s perception of AS as a healthier alternative. Public health strategies should focus on increasing awareness of the potential risks and encourage evidence-based dietary choices.

## 1. Introduction

Artificial sweeteners (AS) are synthetic or naturally derived substances that provide a sweet taste to foods and beverages with little to no caloric value [1]. Common examples include aspartame, saccharin, sucralose, and acesulfame potassium. These compounds are often hundreds of times sweeter than sucrose and are widely used in “diet” or “sugar-free” products to replicate sweetness without the associated energy intake [2]. Their primary function is to serve as sugar substitutes, especially for individuals seeking to reduce overall calorie consumption, manage blood glucose levels, or prevent dental caries. In this regard, AS have been incorporated into dietary strategies aimed at controlling weight and managing conditions such as diabetes mellitus (DM) and metabolic syndrome [3,4].

The popularity of AS has grown significantly in recent years, driven by public health concerns surrounding excessive sugar intake and its strong association with obesity, type 2 diabetes (T2D), and cardiovascular disease (CVD) [5,6]. Individuals pursuing healthier lifestyles or attempting to manage chronic conditions often substitute sugar with AS-containing products due to their non-caloric or low-calorie properties [7,8]. Some studies have suggested that replacing sugar-sweetened beverages with artificially sweetened alternatives may help reduce total sugar intake and overall caloric consumption [9]. Additionally, certain AS, such as aspartame, have been identified as non-carcinogenic and may support oral health by limiting the dietary sugar that contributes to tooth decay [10]. However, despite their widespread use [11,12], the long-term health effects of AS remain controversial and subject to scientific debate [13]. Nevertheless, recent systematic reviews of controlled trials consistently confirm their benefits for weight control, with no robust evidence of adverse health effects [14,15].

As the uncertainty surrounding AS use persists, evidence of potential negative health effects associated with AS consumption has been increasing in recent years. Recent studies have raised concerns about the supposed health benefits of AS [16], with many reporting potential associations between AS use and adverse health outcomes, such as increased risks of obesity [17] and CVD [18]. This complexity surrounding the health implications of AS use emphasizes the need for continued research to clarify their role in human health, weighing potential benefits against emerging evidence of associated risks.

Based on the substantial evidence indicating that AS do not provide beneficial effects for body weight management, the World Health Organization (WHO) has issued a conditional recommendation against the use of AS for weight control or for reducing the risk of non-communicable diseases [19]. Non-communicable diseases, including obesity, T2D, CVD, and cancers, are the critical public health challenges in Hungary [20], where their prevalence ranks among the highest in Europe [21].

Given the rising prevalence of non-communicable diseases and the increasing availability and marketing of artificially sweetened products, it is essential to understand who consumes these sweeteners and under what circumstances. Rather than adopting a definitive stance on the health effects of AS, an investigation focused on usage patterns and consumer characteristics is crucial for evaluating the broader public health implications of AS consumption. Such an approach can help identify vulnerable subpopulations, reveal potential socioeconomic or behavioral disparities, and inform more targeted and equitable public health strategies, especially in light of ongoing debates and mixed scientific evidence surrounding AS safety and efficacy. Accordingly, this study aims to examine the sociodemographic and health-related determinants of AS use in Hungary, drawing on data from the European Health Interview Survey (EHIS)—a nationally representative dataset that captures comprehensive information on health status, lifestyle, and socioeconomic conditions.

## 2. Materials and Methods

### 2.1. Study Design and Data

This study used a cross-sectional approach, drawing on data gathered in Hungary in 2019 through the EHIS. The data were collected using a standardized questionnaire under Eurostat’s supervision, with a sample (*n* = 5603) that was sufficiently representative of Hungary’s general population [22].

### 2.2. Variables

To construct the outcome variable for AS use, we utilized two survey items from the dataset. Participants were classified as AS users if they answered “yes” to at least one of the following: (1) they regularly used AS in tea or coffee, or (2) they consumed at least 250 mL per day of calorie-free, artificially sweetened beverages. Those who did not meet either of these criteria were categorized as non-users. Demographic and socioeconomic variables examined in relation to AS use included gender (male, female), age group (≤18, 18–34, 35–64, ≥65 years), employment status (employer, non-employer, disabled or retired, student or inactive), partner status (lives with a partner, lives alone), household type (one-person household, one parent with child[ren], couple with child[ren]), education level (primary, secondary, high), household income quintile (first [lowest] to fifth [highest]), and degree of urbanization (low, medium, high).

In order to assess how health-related characteristics influence AS use in the population, we analyzed a range of health-related variables, including self-perceived health status, presence of long-term illness or health problems, self-rated oral health, BMI (normal BMI < 25, overweight 25 ≤ BMI ≤ 29.9, obese BMI ≥ 30), mental health status based on WHO-5 index (poor ≤ 50, better > 50), limitations in daily activities due to health problems, regular use of medicines and medicinal preparations and presence of chronic metabolic conditions, including diabetes, hypercholesterolemia, and hypertension.

### 2.3. Statistical Analysis

Descriptive statistics were used to characterize the study population. Categorical variables were summarized using frequencies and percentages. Associations between AS use and categorical socio-demographic and health-related variables were assessed using Pearson’s chi-square test. To identify independent predictors of AS use, we conducted multivariate logistic regression analyses. Odds ratios (ORs) and 95% confidence intervals (CIs) were calculated, adjusting for significant socio-demographic and health-related variables included in the model. Not all health-related variables were included in the final logistic regression model to ensure model stability and avoid multicollinearity. Variable selection was based on a combination of bivariate significance (*p* < 0.05 in chi-square tests), theoretical relevance supported by previous literature, and the assessment of intercorrelations between predictors. Variables showing strong theoretical associations with AS use and statistically significant relationships in unadjusted analyses were prioritized, while highly correlated variables or those with limited added explanatory value were excluded. The predictive accuracy of the model was evaluated using the area under the receiver operating characteristic curve (AUC).

In addition, interaction terms were utilized in a set of three adjusted logistic regression models to assess potential effect modification. These models examined whether the associations between BMI and household income, BMI and age, and age and self-perceived health differed across subgroups based on the associations observed in the main logistic regression model. All statistical analyses were conducted using sampling weights to account for the complex survey design of the EHIS, ensuring representativeness of the population. STATA IC version 18.0 was used to conduct the statistical analyses used in this study [23].

## 3. Results

Table 1 compares the demographic and socioeconomic characteristics of AS users and non-users among 5603 participants, of whom 20.1% reported AS use. While no significant gender differences were observed, AS use was significantly associated with age, employment status, partner status, household type, and income level (all *p* < 0.05). Older adults, retired or disabled individuals, those living with a partner or in households with children, and participants in middle-income quintiles were more likely to report AS use. These findings highlight distinct sociodemographic patterns in sweetener consumption.

Comparisons of health-related characteristics between AS users and non-users are shown in Table 2. AS use was significantly associated with self-perceived general health, oral health, BMI, presence of long-term illness, mental health status (WHO-5), and limitations in daily activities (all *p* < 0.05). Participants reporting poorer general or oral health, chronic health conditions, or limitations in daily functioning were more likely to use AS. A clear gradient was observed with BMI, as AS use increased progressively from normal weight to obesity. Similarly, individuals with lower mental well-being reported higher AS use compared to those with better mental health. These findings suggest that AS consumption is more common among individuals facing physical or psychological health challenges, potentially reflecting efforts to reduce sugar intake or manage health-related risks.

Table 3 presents the results of the logistic regression analysis identifying socio-demographic and health-related factors independently associated with AS use. Older age (≥65 years), higher household income (particularly in the 3rd to 5th quintiles), and higher BMI (overweight and obesity) were significantly associated with increased odds of AS use. Self-perceived health status also showed a significant association, with individuals reporting satisfactory or poor health more likely to consume AS than those in very good health. In contrast, no significant associations were observed for self-rated oral health or mental health status as measured by the WHO-5 index. These results suggest that AS use in Hungary is more prevalent among individuals with greater age, higher income, elevated BMI, and less favorable self-rated health.

Table 4 presents the results of interaction models assessing whether associations between key predictors and AS use were modified by socioeconomic and health-related factors. In Model 1 (BMI × income), the positive association between BMI and AS use was decreased among individuals in the 3rd income quintile, suggesting that overweight and obese individuals in the middle-income group were less likely to use AS compared to their counterparts in the lowest income quintile. Model 2 (BMI × age) found no significant interaction, although the main effect of overweight remained strong. In Model 3 (age × self-perceived health), older adults (≥65 years) with good health were significantly less likely to use AS than those of the same age reporting poorer health. No significant interactions were observed in the remaining models (BMI × health status, income × health status, income × age). These findings indicate that socioeconomic context and self-rated health may influence the strength of associations between BMI, age, and AS use.

## 4. Discussion

In this study, we aimed to identify the socio-economic, demographic, and health factors influencing AS use among the Hungarian population. The results revealed that older age, people with an upper-middle income, those who are overweight or obese, and those who perceive their health as poor are more likely to use AS.

Results from previous studies show complex patterns in AS use and key demographic variables such as age and gender. Women consume AS more than men and face higher risks of hypertension [24] and gestational diabetes during pregnancy [25]. Additionally, women are more aware of potential adverse effects, influencing dietary choices [18], and face distinct cardiovascular risks [26]. No significant gender differences in AS consumption were found in Hungary, possibly due to unique dietary traits of the population. However, in terms of age, it was found that older people are more likely to use AS in our results. Research indicates that older adults are increasingly becoming consumers of AS. For instance, Alharthi et al. found that the age group of those using AS has shifted, with a significant increase in older adults participating in such consumption patterns [12]. This trend has been attributed to a heightened awareness of health risks associated with sugar intake, leading older adults to seek alternatives in an effort to manage weight and reduce health complications. Comparatively, younger populations, particularly university students, display different consumption patterns, and a study reported that approximately 30% of university students consumed AS, detailing a prevalent trend among younger adults in managing body weight and health through diet [27]. This suggests that younger people are more likely to use AS for weight control, while older people are more likely to use AS for health reasons, such as to prevent obesity, T2D, and the risk of CVD and its complications, which in either case contradicts WHO recommendations.

It has been established that socio-economic status strongly influences the use of AS. A study demonstrates that individuals from lower socioeconomic backgrounds tend to consume higher amounts of sugar-sweetened beverages, which is often related to the affordability and accessibility of lower-cost, calorie-dense foods [28]. Similarly, an earlier study noted that lower-income populations are often targeted by aggressive marketing campaigns for both sugar-sweetened and artificially sweetened beverages, which could contribute to higher consumption rates in these groups [29]. It suggests that the lower nutritional knowledge and education levels among lower socioeconomic status groups may exacerbate these consumption patterns. A systematic review has highlighted the critical role of food prices in influencing dietary quality across socioeconomic groups. It found that individuals with higher socioeconomic status tend to spend more on food and adopt higher-quality dietary patterns, which may reduce their use of AS. In contrast, those with lower incomes often face restricted access to nutritious yet costly foods, leading to greater dependence on inexpensive, lower-quality products, including those with AS, either for perceived health advantages or weight control [30]. Interestingly, while previous literature suggests that individuals with lower income are more likely to consume artificially sweetened products, our findings showed that AS use was actually higher among those in higher income groups. However, when examining the interaction between BMI and income, we found that overweight and obese individuals in the middle-income group were less likely to use AS compared to those in the lowest-income group. This suggests that among individuals with excess weight, those with the lowest income may be more motivated to use AS, possibly due to increased health concerns or exposure to targeted marketing promoting AS as a calorie-reducing option. In contrast, AS use among higher-income individuals may reflect broader lifestyle choices or health-conscious behavior rather than income itself being the primary factor. These findings underscore the importance of considering both socioeconomic position and health status when analyzing AS consumption patterns.

AS have long been used as calorie-free alternatives to sugar, particularly in the context of weight management and glycemic control. This usage has influenced consumer behavior and dietary recommendations, especially for populations at risk of obesity and related metabolic conditions. Our findings are consistent with previous research examining these associations [31], which found that individuals with overweight or obesity, who are often targeted by AS marketing and dietary recommendations, are more likely to consume AS products. This suggests that AS use may be perceived as a practical strategy to reduce sugar intake or limit caloric consumption in the context of managing body weight. However, while AS are widely used with the intention of supporting healthier dietary patterns, their long-term health effects remain a subject of ongoing scientific debate. Recent systematic reviews and meta-analyses have produced mixed findings. Some studies report modest benefits, including short-term reductions in body weight and energy intake when AS are used to replace sugar-sweetened products [32]. Conversely, other reviews raise concerns about possible adverse health outcomes, including associations with weight gain, insulin resistance, and increased risk of CVD [33]. Adding to this complexity, newer evidence suggests that different AS compounds may exert varied biological effects. Emerging studies point to potential mechanisms by which certain AS may contribute to systemic inflammation, impair glucose metabolism, and disrupt gut microbiota—factors that are themselves associated with increased BMI and obesity [34]. These mechanistic pathways may partially explain the inconsistent outcomes observed across epidemiological studies and clinical trials. In light of this uncertainty, the WHO has recently issued a conditional recommendation discouraging the use of AS for body weight control or the prevention of non-communicable diseases [19]. This guidance reflects a growing recognition that while AS may offer short-term substitution benefits, their long-term implications for metabolic health remain inconclusive and potentially problematic.

Besides obesity, the use of AS is also driven by the presence of certain health conditions and self-perceived health status, as shown in our results. Several studies indicate that individuals with existing health conditions, particularly diabetes and CVD, often use AS, complicating the understanding of their health implications. A study focused on patients with diabetes found a significant relationship between daily consumption of AS and the prevalence of hypertension among these individuals. While the molecular mechanisms linking AS to hypertension remain unclear, their findings suggest that AS may pose cardiovascular risks, elevating health concerns within this population [35]. This aligns with other studies indicating potential increased risks of CVD associated with AS consumption across various demographics [25,36]. A recent review concluded that excessive intake of AS has been linked to various health risks, including metabolic disorders, cardiovascular diseases, and gut microbiome disruption [36].

Even though the main mechanisms behind the associations between health risks and AS use remain unclear, various studies have proposed different explanations. First of all, AS may disrupt the body’s ability to associate sweetness with caloric intake, potentially leading to overeating and weight gain [37]. Furthermore, with long-term use, AS increases the risk of developing insulin resistance and nonalcoholic fatty liver disease [38]. This is supported by evidence that commonly used sweeteners such as aspartame and sucralose have been shown to negatively impact metabolic health [39]. AS use may also alter gut microbiota composition, leading to dysbiosis and increased risk of T2D [40]. In addition, AS may promote inflammation and oxidative stress, contributing to cardiovascular and metabolic issues [41,42]. Although findings are still debated, some studies raise concerns about the potential carcinogenic effects of certain AS [43].

In light of these findings and the WHO’s conditional recommendation discouraging the use of AS for body weight control and prevention of non-communicable diseases, there is a need for stronger national policy guidance in Hungary. Public health authorities could consider incorporating WHO recommendations into national dietary guidelines and launching awareness campaigns to address misconceptions about the health benefits of AS. Additionally, front-of-package labeling regulations could be updated to clearly indicate the presence of artificial sweeteners, helping consumers make informed choices. Education efforts targeting high-risk groups such as older adults and individuals with obesity or diabetes could be especially impactful. These actions would help bridge the current policy gap and align Hungary’s dietary practices more closely with global public health guidance.

This study has several strengths. It uses nationally representative data from the EHIS, which ensures broad generalizability to the Hungarian population. The large sample size and inclusion of a wide range of sociodemographic and health-related variables allow for a comprehensive analysis of factors associated with AS use. Additionally, the use of weighted logistic regression and interaction models strengthens the validity of the findings by accounting for complex survey design and exploring nuanced relationships between variables. However, several limitations should be noted. The cross-sectional nature of the study limits causal inference between AS use and associated factors. Self-reported data may be subject to recall or social desirability bias. Furthermore, detailed variables on AS use, including type, duration, or frequency of AS, were not available in the EHIS dataset, which may have led to residual confounding.

## 5. Conclusions

Our results demonstrate that adults aged 65 years and older, individuals in higher income quintiles, those who are overweight or obese, and those reporting poor self-perceived health were significantly more likely to use AS, highlighting socioeconomic and health-related disparities in AS consumption within the Hungarian population. Interaction analyses revealed that overweight and obese individuals in the lowest income group were more likely to use AS compared to those in the middle-income group. This suggests that body weight, rather than income, may be the main reason for AS use. Similarly, older adults in poor health were more likely to use AS than their healthier counterparts. These findings contrast with the WHO guideline, which advises against the use of AS for body weight control and the reduction of non-communicable disease risk. This discrepancy indicates a potential gap in public knowledge or misperception regarding the health benefits of AS, underscoring the need for better-informed public health messaging.

Public health interventions should focus on improving awareness of the limitations and potential risks of AS use, especially among high-risk groups, and promote evidence-based strategies for weight and disease management. Furthermore, longitudinal research is needed to better understand the causal impact of AS consumption on health outcomes, including detailed use patterns and dose responses. Qualitative research on consumer views could also help tailor better public health messages and policies.

## Figures and Tables

**Table 1:** Comparison of demographic and socioeconomic variables by AS use.

Variables	Category	AS Usage, *n* (%)		*p* Value
		Non-AS Users	AS Users	
Gender	Male	2071 (80.5)	501 (19.5)	0.261
	Female	2404 (79.3)	627 (20.7)	
Age group	≤18	159 (89.3)	19 (10.7)	**<0.001**
	18–34	928 (84.5)	170 (15.5)	
	35–64	2183 (80.9)	516 (19.1)	
	≥65	1205 (74.0)	423 (26.0)	
Employment status	Employer	2303 (82.1)	503 (17.9)	**<0.001**
	Non-employer	410 (83.2)	83 (16.8)	
	Disabled or Retired	1446 (74.2)	504 (25.2)	
	Student or Inactive	316 (89.3)	38 (10.7)	
Partner status	Lives with a partner	2460 (78.4)	678 (21.6)	**0.002**
	Lives alone	1910 (81.9)	423 (18.1)	
Type of household	One-person household	1822 (81.9)	403 (18.1)	**0.009**
	One parent with child(ren)	283 (78.4)	78 (21.6)	
	Couple with child(ren)	2368 (78.5)	647 (21.5)	
Education levels	Primary	973 (80.8)	231 (19.2)	0.084
	Secondary	2481 (78.8)	666 (21.2)	
	High	1021 (81.6)	231 (18.4)	
Quintiles based on net equivalent household income	First (lowest)	985 (83.1)	195 (16.1)	**0.012**
	Second	931 (79.4)	242 (20.6)	
	Third	880 (77.3)	259 (22.7)	
	Fourth	1019 (80.3)	250 (19.7)	
	Fifth (highest)	687 (79.9)	182 (20.1)	
Degree of urbanization	Low	1458 (79.8)	368 (20.2)	0.339
	Medium	1470 (78.9)	393 (21.1)	
	High	1547 (80.8)	367 (19.2)	

Bold values indicate statistical significance (*p* < 0.05) based on Chi-square test.

**Table 2:** Comparison of health-related variables by AS use.

Variables	Category	AS Usage, *n* (%)		*p* Value
		Non-AS Users	AS Users	
Self-perceived health status	Very good	774 (85.6)	130 (14.4)	**<0.001**
	Good	1890 (82.9)	391 (17.1)	
	Satisfactory	1312 (74.5)	450 (25.5)	
	Bad	364 (74.9)	122 (25.1)	
	Very bad	104 (75.9)	33 (24.1)	
Long-term illness or health problems	Yes	2082 (74.4)	716 (25.6)	**<0.001**
	No	2292 (85.2)	339 (14.8)	
Self-rated oral health	Very good	459 (83.3)	92 (16.7)	**<0.001**
	Good	1684 (82.4)	361 (17.6)	
	Satisfactory	1428 (78.0)	402 (22.0)	
	Bad	647 (76.3)	201 (23.7)	
	Very bad	222 (77.1)	66 (22.9)	
Body mass index	Normal (BMI < 25)	1870 (84.8)	335 (15.2)	**<0.001**
	Overweight (25 ≤ BMI < 30)	1511 (77.9)	428 (22.1)	
	Obese (BMI > 30)	1031 (74.0)	362 (26.0)	
Mental health status	Poor (WHO-5 index ≤ 50)	963 (77.3)	283 (22.7)	**0.010**
	Better (WHO-5 index > 50)	3512 (80.6)	845 (19.4)	
Health problems that limit daily activities	Yes, severe problems	306 (72.9)	114 (27.1)	**<0.001**
	Yes, but not severe	941 (76.3)	292 (23.7)	
	No	3185 (81.7)	712 (18.3)	
Regular use of medicines and medicinal preparations	Prescribed and non-prescribed medicines	1360 (73.6)	487 (26.4)	**<0.001**
	Only prescribed medicines	914 (75.5)	296 (24.5)	
	Only non-prescribed medicines	946 (84.8)	169 (15.2)	
	Do not use medicines	1221 (87.5)	174 (12.5)	
Diabetes mellitus	No	4150 (83.2)	838 (16.8)	**<0.001**
	Yes	263 (48.3)	282 (51.7)	
Hypercholesterinemia	No	3804 (81.3)	877 (18.7)	**<0.001**
	Yes	555 (71.1)	226 (28.9)	
Hypertension	No	2992 (83.4)	594 (16.6)	**<0.001**
	Yes	1140 (73.1)	530 (26.9)	

Bold values indicate statistical significance (*p* < 0.05) based on Chi-square test.

**Table 3:** Weighted logistic regression results for AS use in the Hungarian population.

Variables	Odds Ratio	95% CI (Lower)	95% CI (Upper)	*p* Value
Age group (Reference: <35 years old)				
35–64 years old	1.104	0.894	1.364	0.358
≥65 years old	1.434	1.134	1.814	**0.003**
Income level (Reference: 1st quintile)				
2nd quintile	1.183	0.942	1.487	0.148
3rd quintile	1.446	1.149	1.819	**0.002**
4th quintile	1.263	1.003	1.589	**0.047**
5th quintile	1.570	1.217	2.025	**0.001**
BMI (Reference: Normal)				
Overweight	1.421	1.191	1.695	**<0.001**
Obese	1.761	1.461	2.122	**<0.001**
Self-perceived health (Reference: Very good)				
Good	1.143	0.890	1.468	0.295
Satisfactory	1.516	1.151	1.995	**0.003**
Bad	1.549	1.088	2.206	**0.015**
Very bad	1.267	0.762	2.107	0.361
Self-reported oral health (Reference: Very good)				
Good	0.945	0.713	1.251	0.692
Satisfactory	1.019	0.758	1.370	0.899
Bad	1.104	0.792	1.539	0.560
Very bad	1.063	0.704	1.604	0.773
Mental health status (Reference: Poor)				
Better mental health	0.905	0.757	1.080	0.268

Bold values indicate statistical significance (*p* < 0.05). Odds ratios are adjusted for variables in the model. AUC = 0.612 (95% CI 0.601–0.637).

**Table 4:** Logistic regression models predicting AS use with interaction terms.

Interaction Term	Model	OR	95% CI	*p*-Value
Overweight X 3rd Income Quintile/1st Income Quintile	Model 1	0.58	0.35–0.98	**0.043**
Obese X 3rd Income Quintile/1st Income Quintile	Model 1	0.56	0.33–0.95	**0.030**
Overweight X Age ≥ 65/<35 years old	Model 2	0.64	0.41–1.01	0.057
Obese X Age ≥ 65/<35 years old	Model 2	1.15	0.67–1.99	0.637
Age ≥ 65 X Good Health/Bad Health	Model 3	0.39	0.18–0.86	**0.019**

OR = Odds ratio; 95% CI = 95% confidence interval. Model 1: Interaction between body mass index (BMI) and household income; Model 2: Interaction between BMI and age; Model 3: Interaction between age and self-perceived health status. All models are adjusted for the same variables in the weighted logistic regression model shown in Table 3. Bold values indicate statistical significance (*p* < 0.05) based on interaction terms.

## Data Availability

The data analyzed in this study is subject to the following licenses/restrictions: The data presented in this study are available upon request from Hungarian Central Statistical Office, who performed and supervised the data collection. Requests to access these datasets should be directed to Hungarian Central Statistical Office, www.ksh.hu/?lang=en (accessed on 25 May 2025).
